# Impact of a Single Phage and a Phage Cocktail Application in Broilers on Reduction of *Campylobacter jejuni* and Development of Resistance

**DOI:** 10.1371/journal.pone.0078543

**Published:** 2013-10-21

**Authors:** Samuel Fischer, Sophie Kittler, Günter Klein, Gerhard Glünder

**Affiliations:** 1 Clinic for Poultry, University of Veterinary Medicine, Hannover, Lower Saxony, Germany; 2 Institute of Food Quality and Food Safety, University of Veterinary Medicine, Hannover, Lower Saxony, Germany; The University of Melbourne, United States of America

## Abstract

Campylobacteriosis is currently the most frequent foodborne zoonosis in many countries. One main source is poultry. The aim of this study was to enhance the knowledge about the potential of bacteriophages in reducing colonization of broilers with *Campylobacter* , as there are only a few *in vivo* studies published. Commercial broilers were inoculated with 10^4^ CFU/bird of a *Campylobacter jejuni* field strain. Groups of 88 birds each were subsequently treated with a single phage or a four-phage cocktail (10^7^ PFU/bird in CaCO_3_ buffered SM-Buffer). Control birds received the solvent only. Afterwards, subgroups of eleven birds each were examined for their loads with phages and *Campylobacter* on day 1, 3, 7, 14, 21, 28, 35 and 42 after phage application. The susceptibility of the *Campylobacter* population to phage infection was determined using ten isolates per bird. In total 4180 re-isolates were examined. The study demonstrated that the deployed phages persisted over the whole investigation period. The *Campylobacter* load was permanently reduced by the phage-cocktail as well as by the single phage. The reduction was significant between one and four weeks after treatment and reached a maximum of log_10_ 2.8 CFU/g cecal contents. Phage resistance rates of initially up to 43% in the single phage treated group and 24% in the cocktail treated group later stabilized at low levels. The occurrence of phage resistance influenced but did not override the *Campylobacter* reducing effect. Regarding the reduction potential, the cocktail treatment had only a small advantage over the singe phage treatment directly after phage administration. However, the cocktail moderated and delayed the emergence of phage resistance.

## Introduction

Human campylobacteriosis is presently the most frequent foodborne zoonosis in the EU with stable rates of about 53 confirmed cases per 100 000 population and year during 2006-09 [1]. Campylobacteriosis is a diarrheal disease with symptoms of severe abdominal pain, watery and/or bloody diarrhea and fever, after an incubation period of 2–5 days. Usually the disease is self-limiting, but it occasionally results in hospitalization. Antimicrobial therapy is seldom needed. *Campylobacter* infection has been associated with sequelae such as joint inflammation and Guillain-Barré- and its variant Miller-Fisher-syndrome [2]. The most frequently reported *Campylobacter* (C.) species are *C. jejuni*, *C. coli* and *C. lari* [1]. *Campylobacter* *spp.* can only multiply in warm blooded animals such as poultry, cattle, pigs, wild birds and wild mammals and are excreted into the environment. One main cause of human campylobacteriosis is handling, preparation and consumption of broiler meat, which may account for 20% to 30 percent of human infections with *Campylobacter*. A total of 50% to 80 percent of human infections are thought to be attributed to chicken farming [3]. A reduction of intestinal colonization of broilers would lead to a considerable decline of human campylobacteriosis [4]. A risk assessment showed that a reduction of log_10_ 2 of *Campylobacter* counts on broiler carcasses leads to a 30-fold decline in human campylobacteriosis caused by consuming chickens [5]. Currently available conventional pre-harvest strategies to reduce *Campylobacter* contamination in poultry production are not sufficient [6]. These pre-harvest strategies include the application of on-farm biosecurity measures, the decontamination of litter, and the supplementation of feed with compounds inhibiting *Campylobacter* and the treatment of drinking water. Moreover, novel strategies, specifically targeting *Campylobacter* control at pre-harvest level, are in progress, including administration of probiotics, vaccination, antibiotics, changes in feed structure and antimicrobial alternatives, i.e. bacteriophages (phages) and bacteriocins [7,8]. Bacteriophages are ubiquitous in the environment and appear naturally on food for human consumption along with their target bacteria [9–12]. A reduction of *Campylobacter* counts by an average of log_10_ 2 and a maximum of log_10_ 5 was demonstrated by using phages in several *in vivo* studies [13–16]. Phages are very specific to their target host having little impact on the remaining microorganisms. Thus, phages are a promising supplementary tool for the production of safe poultry food products [17].

The usage of phages for farm animals is of particular interest because it may help to reduce the administration of antibiotics. Antibiotic medication only leads to a temporary reduction, but not to an elimination of *Campylobacter* [18] and attracts long-lasting public criticism regarding increasing bacterial resistance. Unsurprisingly, after phage treatment, the development of phage resistant *Campylobacter* strains has been observed. Resistance rates from 2% [9] up to 13% [8] have been found. Loc Carrillo et al. [13] observed that a phage resistant *Campylobacter* isolate had a significantly reduced ability to colonize the broiler intestine compared to the original susceptible strain. Carvalho et al. [8] did not observe the same effect. Questions remain concerning the preservation of resistance during further intestinal passage, which may lead to loss of the resistant phenotype. In order to avoid the accumulation of phage resistant *Campylobacter* strains in the environment Goode et al. [19] suggested limiting the use of phages to the slaughter house as an epidemiological endpoint. In contrast, Wagenaar et al. [15] considered the release of phages in the environment to be acceptable and proposed a rotating use of different phages in order to deal with resistance problems.

We tested phage bio-control under controlled conditions in broilers over six weeks with particular regard to the development of resistances against the phages, comparing a four-phage cocktail and a single phage treatment.

## Materials and Methods

### Ethical statement on bird experiment

This study was carried out in strict accordance with the recommendations in the Guide for the Care and Use of Laboratory Animals of the National Institutes of Health. The use of animals in this study was approved by the Animal Welfare Officer of the University of Veterinary Medicine, Foundation, Hannover, whose tasks include the scrutiny of animal welfare, ethics and handling, and then announced to the Lower Saxony State Office for Consumer Protection and Food Safety in accordance with §8a(1,2) of the German Animal Health and Welfare Act. The Lower Saxony State Office for Consumer Protection and Food Safety approved the work on this study under permit number 33.9-42502-05-11A153. The study was notifiable but not subject to approval according to §8(7) Nr. 2 of the German Animal Health and Welfare Act. The accomplishment of the experiment was supervised by a competent person in accordance with the §9 of the German Animal Health and Welfare Act to ensure the compliance of §9 and §9a of the German Animal Health and Welfare Act and all efforts were made to minimize animal suffering.

### Bacteriophages

#### General information

The phages 1, 2, 5 and 13 (table 1) from the British phage typing scheme were used for this study. Propagation of phages and determining concentration of phage suspensions were performed as previously described [20]. Phage suspensions were stored at 4°C. 

**Table 1:** Phage strains and their *Campylobacter* host strains.

No of Phage strains			*Campylobacter* strains (NCTC^1^-No.)	
Phage	NCTC^1^		propagation	determination of concentration
1	12673		12661	12662
2	12674		12661	12662
5	12678		12664	12662
13	12672		12660	12662

1 National Collection of Type Cultures

#### Phage treatment

Phage suspensions of each phage were diluted in SM-buffer (5.8g NaCl, 2.0g MgSO_4_x7H_2_O, 50ml 1M Tris (Sigma) pH 7.5, 5ml 2% gelatine, filled up with distilled water to 1000ml) to approximately 10^7^ PFU/ml, mixed in equal parts for the cocktail application and supplemented with 33% (w/v) CaCO_3_ (Roth, 6230.1). Subsequently 1 ml of the suspension was administered directly into the crop of each broiler.

#### Isolation of phages from cecal contents

1g of cecal contents was mixed thoroughly 1:10 with the SM- buffer and shaken overnight at 4°C. Chloroform (Sigma-Aldrich, 366919) was added to the sample at a concentration of 5% (v/v), which was then shaken at 300rpm for 15 min and subsequently centrifuged at 13,000 x g for 20 min. The phage containing supernatant was filtered through a 0.22 µm filter (Roth, P668.1) to eliminate bacterial compounds and then serially diluted in the SM- buffer for determining the phage concentration.

### Campylobacter

#### General information

All *Campylobacter* strains were cultured and stored in several aliquots at -70°C to serve as a master seed before studies commenced. Laboratory *Campylobacter* strains, used for propagating phage strains and determining phage concentration are listed in table 1. *Campylobacter jejuni* 1474-06 was used as the model field strain for all experiments.

*Campylobacter* was always cultivated under microaerobic conditions (Oxoid Gas Pak System, Campygen) at 42±0.5°C.

Mc-Farland-Standards (McFSt.) of *Campylobacter* strains were prepared for propagating phages or determining phage concentrations as well as for susceptibility and resistance testing of *Campylobacter* isolates. A single colony of the required strain or respective isolate was plated on Mueller-Hinton-Agar (Oxoid) and incubated for 16h. The bacteria were then harvested with a sterile cotton swab, dispensed in 10 mmol MgSO_4_ solution and adjusted to the McFSt. needed for the particular application in a Densimat (Biomerieux SA France, IDN 013615).

#### Model organism

The model organism *C. jejuni* strain 1474-06 was isolated in an abattoir from poultry pectoral muscles in 2006. This strain is representative for a collection of well characterized *Campylobacter* field strains and susceptible to all phages used in the United Kingdom Typing Scheme except phage 12 (NCTC12677) [21]. 

For inoculation of broilers *Campylobacter* 1474-06 was propagated in Standard 1 nutrient broth (Merck) for 48h and subsequently diluted to approximately 10^6^ CFU/ml. Reisolation was performed on Karmali Agar (Oxoid). For this, serial dilutions of 1g of cecal contents per broiler were made in phosphate buffered saline (8g NaCl, 2g KH_2_PO_4_, 2.9g Na_2_HPO_4_, H_2_O ad 1l, adjusted to pH 7.4 with HCl/NaOH). Aliquots of 100µl were plated on Karmali Agar, from each dilution two separate plates were made. The colonies counted after two days incubation allowed the calculation of the CFU/g cecal contents.

#### Susceptibility testing

The susceptibility of the re-isolated *Campylobacter* 1474-06 strains for each single phage and for the cocktail was tested as previously described [20]. This test distinguishes between isolates that are as susceptible as the original strain and those, that have reduced susceptibility or are resistant.

#### Resistance testing

Resistance of *Campylobacter* 1474-06 and re-isolated strains against the phages was examined using the conventional agar overlay plate assay: a suspension of each *Campylobacter* strain was adjusted to McFSt. 3. NZCYM-overlay-agar (NZCYM broth 22g/l, Roth X974.1, Agar-agar 7g/l, Roth 2266.3) was liquefied in tubes and kept molten at 48±0.5°C in a block heater (Roth, Rotalibo-Block-Heater H250). 100µl of the *Campylobacter* suspension and 100µl of the test phage-suspension, containing 10^4^ PFU/ml, were added to 5ml NZCYM-overlay-agar, mixed thoroughly and poured into a petri dish containing 20 ml NZCYM-base (NZCYM-broth 22g/l, Agaragar 15 g/l). After the overlay had solidified, plates were inverted and incubated at 42±0.5°C for 24 hours.

### Housing and care of laboratory animals

Broilers were used as an animal model, due to the fact that the study was aimed at reducing *Campylobacter* in commercial broiler production. Each group of birds was placed in separate units of the clinic-affiliated isolation facility as day-old chicks and each was cared for separately by one specialized animal keeper to avoid any environmental and cross contamination. The birds received commercial complete feed and water from the municipal water supply in bell drinkers ad libitum. The broilers were kept on wood shavings approximately 2kg/m^2^. The temperature was in accordance with commercial rearing conditions. The stocking density was 13 individual birds per square meter at the beginning and reduced by the taking of broilers for necropsy to 1.6 birds/m² at the end of the experiment.

### Trial design

#### General experimental setup

92 Commercial broilers per group were housed as day-old chicks. On day six of life, four birds per group were sacrificed in order to confirm the birds were *Campylobacter* negative via culture. The other birds received 1ml of *Campylobacter* suspension, adjusted to 10^4^ CFU/ml, directly into the crop. After three days of *Campylobacter* colonization (day nine of life), each bird of the treatment groups received 1 ml CaCO_3_-buffered phage suspension, containing 10^7^ PFU/ml, directly into the crop. The phage cocktail was composed of phages 1, 2, 5 and 13 in equal parts. On day 10, 12, 16, 23, 30, 37, 44 and 51 of life, eleven birds per group were sacrificed. Before the dissection the birds were weighed. Ceca were removed aseptically and immediately processed within the clinic-affiliated laboratories. Approximately 1 g of cecal contents from each bird was weighed out twice. The first sample was diluted 1:10 in PBS, the second in SM-buffer then both were mixed thoroughly. Serial dilutions of these suspensions were made in order to determine the concentration of *Campylobacter* and phages as described above. Single *Campylobacter* colonies were picked for examination of phage susceptibility.

#### Trial I

The treatment group received the buffered phage cocktail. The control group received the buffer suspension only. Ten re-isolated *Campylobacter* colonies per bird were tested for phage susceptibility. Thus, one hundred colonies from ten birds were investigated at each examination date from the treatment and the control group, respectively. These isolates were identified as belonging to the species *C. jejuni* by testing one *Campylobacter* colony from each broiler by PCR [22]. However, testing one colony from one bird cannot confirm that the bird did not harbour other *Campylobacter* spp.. The PCR was thought as a supplementary tool for controlling the biosecurity measures: If a *Campylobacter* strain enters a flock, it rapidly spreads across the flock. Therefore, we regarded each group as one unit, which enhanced the significance. 

#### Trial II

The first treatment group received the buffered phage cocktail. The second treatment group received a buffered suspension, containing phage 1 only. The control group received the phage free buffered suspension medium only. Ten re-isolated *Campylobacter* colonies per bird were tested for phage resistance as described above. Colonies from the cocktail treated group were tested for resistance against each single phage separately and against the phage cocktail. Colonies from the single phage treated group were tested for resistance against phage 1 and also against the phage cocktail. Colonies from the control group were tested for resistance against the cocktail only. 

### Data handling/Calculations

#### Data evaluation

Data evaluation and figure preparation were performed in Microsoft Excel 2010. Significances were calculated using T-Test and Man-Withney Rank Sum Test in Sigma Stat 3.1.

#### SMean

A susceptibility approximate value, in the following named susceptibility mean (SMean) was calculated for each *Campylobacter* colony (SMean_C_), examined for susceptibility or resistance, in order to allow a broad comparison of the groups with the two different phage treatments as well as susceptibility test procedures. The SMean subserves the analysis of interrelations between the phage susceptibility of the *Campylobacter* population on the one hand and the colonization level of *Campylobacter* on the other hand.

SMean_C_ **=** sum of efficacious phages / sum of phages tested

*Campylobacter* isolates from trial I were examined for their susceptibility to each single phage and additionally the phage cocktail in the Microplate-Test. Therefore, isolates which were as susceptible as the original strain were entered as “susceptible” into the calculation, whereas reduced susceptible or resistant isolates were not entered. For trial I a SMean_C_ of “1” was assigned for *Camylobacter* isolates, which were as susceptible as the original strain to each phage. A SMean_C_ of “0” was assigned for *Campylobacter* isolates with reduced susceptibility to each phage.

Accordingly, the SMean was determined for *Campylobacter* isolates from trial II, which were examined for resistance in the conventional agar overlay plate assay. Here, reduced susceptible isolates were not separately detected and entered as “susceptible” in the calculation. Therefore, an SMean_C_ of “1” means, that the appropriate isolate has no resistance against any phage and an SMean_C_ of “0” means, that the isolate is fully resistant to each phage examined.

SMeans, used in this paper, were always calculated for the whole *Campylobacter* population of each single broiler (SMean_B_), whereby the *Campylobacter* population was represented by ten colonies from each bird examined for susceptibility or resistance. 

Therefore, for both trials the general interpretation on bird level of SMean_B_ of “1” is: no resistance against any phage in any isolate from this bird was found. An SMean_B_ of “0” means: all ten isolates from this bird showed at least reduced susceptibility or were even resistant to each phage.

#### Ratio of susceptible and less susceptible/resistant subpopulation

For each individual bird “B”, the extent of the susceptible *Campylobacter* subpopulation was calculated, multiplying the *Campylobacter* load “L_B_” and the corresponding SMean_B_.

extent of susceptible subpopulation (CFU/g) = L_B_ (CFU/g) x SMean_B_

The extent of the less susceptible (trial I) or resistant (trial II) *Campylobacter* subpopulation of any bird was calculated, subtracting its susceptible *Campylobacter* population from its total *Campylobacter* load.

extent of resistant subpopulation (CFU/g)**=** L_B_ (CFU/g) - ( L_B_ (CFU/g) **x** SMean_B_ )

#### Significance of the interpretation of SMean

As the rate of resistant or less susceptible isolates was unknown when the experiments were planned, we carefully considered the costs and benefits of the amount of isolates for investigations and decided to examine ten isolates per bird: If all ten isolates from one bird are susceptible we can say that this bird harbors less than 20% resistant isolates with a safety level of 90%. Regarding the dissection group as one unit under the same conditions we can approximately say that the group harbored fewer than 2% resistant isolates.

## Results

### Phage load

#### Treatment groups

**Figure 1:**
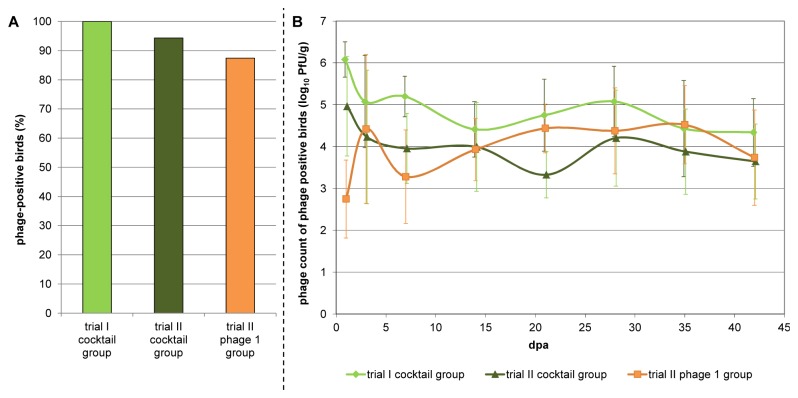
Reisolation of phages. Part A shows the percentage of phage positive birds for each treated group. Part B shows the mean phage count (log_10_) of the positive birds for each treated group. dpa = days post phage application.

The phage counts in cecal contents were determined for each bird. The rate of phage positive birds from each treatment group and the chronological sequence of the mean phage count from these phage positive birds are shown in Figure 1. 

All broilers (100%) in trial I were positive for phages in their cecal contents (Figure 1a). In trial II, treated with the same phage cocktail, five broilers tested negative towards the end of the examination period resulting in a carrier rate of 92%. In the group treated with 1 phage in trial II at least one individual was found to be negative for phages at nearly every examination, leading to a rate of 87% phage-positive birds.

The mean phage titers and standard deviations of the phage treated groups during the course of the experimental trials are shown in Figure 1b: Although all groups were inoculated with log_10_ 7.0 PFU per bird, the cocktail treated groups evolved a mean titer of log_10_ 6.1 (trial I) and log_10_ 5.0 (trial II) PFU/g one day after application in contrast to the single phage treated group, which showed a drop to log_10_ 2.7 PFU/g after one day. From day three after phage application onwards the mean phage titers in all three treated groups altered between log_10_ 3.2 and log_10_ 5.2 PFU/g and all groups leveled off at about log_10_ 4 PFU/g towards the end of the examination period.

The phage counts from the cocktail treated group of trial I, amounting to log_10_ 4.9 PFU/g cecal contents in the mean were higher compared to the counts of the cocktail treated group of trial II, which resulted in log_10_ 3.8 PFU/g. The mean phage count of the phage 1 treated group totaled log_10_ 3.4 PFU/g.

The mean phage titer of all phage treated birds was log_10_ 4.05 PFU/g cecal contents. The highest phage count per bird of log_10_ 7.1 PFU/g was found in the single phage treated group. The highest count per bird in the cocktail treated groups accounted for log_10_ 6.7 PFU/g. 

#### Infected control group of trial I

**Figure 2:**
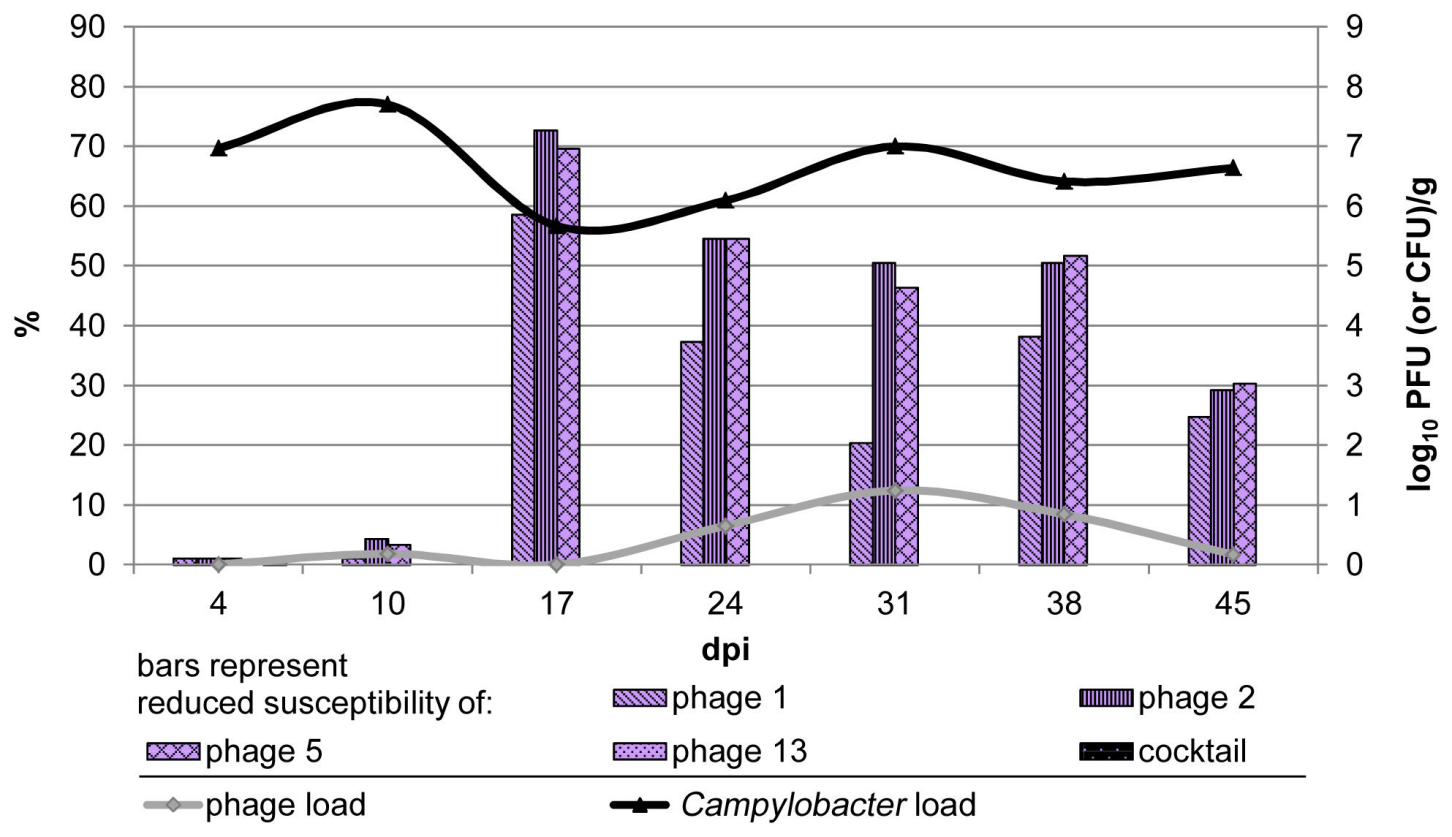
*Campylobacter* counts, phage counts and bacterial phage susceptibility of the unintentional phage infected control group of trial I. The bars show the percentage of *Campylobacter* isolates that were less susceptible to the represented phages. The lines show the corresponding *Campylobacter* counts and the unwanted phage infection. The detection of phages coincided with loss of phage susceptibility and a decrease of *Campylobacter* counts in the control group of trial I.

Phages were isolated from the untreated control group of trial I. The grey line in Figure 2 shows the mean phage counts. The highest count of log_10_ 1.2 PFU/g in the mean value was found in week four (highest count per bird: 1.9 PFU/g ), but did not reach the phage level found in the treatment groups during the investigation period. The mean phage count of the positive birds remained stable at about log_10_ 1 PFU/g, altogether only 32% of all birds were positive. Therefore, the height of the grey line mirrors the proportion of these phage positive birds at the different time points. Once phages were isolated from single birds, the whole group was excluded as control. The control group of trial II could be utilized instead, since the experimental setup was identical in both trials.

### *Campylobacter* load

**Figure 3:**
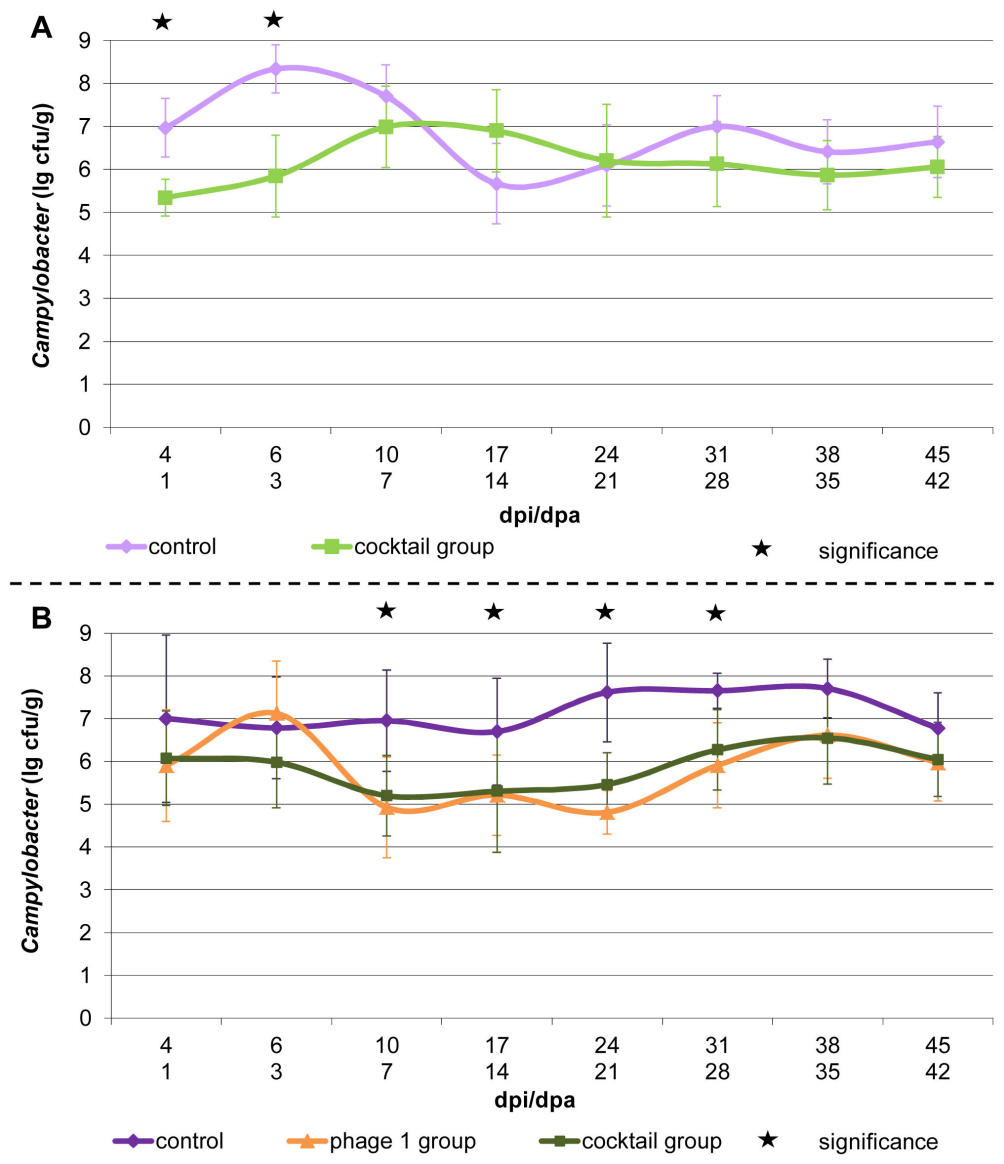
*Campylobacter* counts in ceca. Part A shows the mean *Campylobacter* counts observed in the groups of trial I. Part B shows the mean *Campylobacter* counts observed in the groups of trial II. dpi = days post *Campylobacter*-inoculation dpa = days post phage application.

All re-isolated colonies examined by PCR belonged to the species *Campylobacter jejuni*. The chronological sequence of the mean *Campylobacter* counts from each group is presented separately for trial I (Figure 3a) and trial II (Figure 3b). The *Campylobacter* level in the cecal contents from the individual birds of both trials varied considerably from log_10_ 3.4 CFU/g to log_10_ 9.5 CFU/g. Altogether, the mean *Campylobacter* load of all phage treated birds in this study was log_10_ 5.9 CFU/g cecal contents and is therefore significantly (p<0.001) below the *Campylobacter* load of the phage free control birds from trial II, which was in mean log_10_ 7.2 CFU/g. Both the cocktail and the single phage treatment resulted in an average reduction of log_10_ 1.3 CFU/g in trial II.

In trial I the phage-treatment led to a significantly (p< 0.001) reduced *Campylobacter* load up to three days after phage application (Figure 3a). Subsequently, there was no significant difference between the treatment group and the control along with an infection of the control by low numbers of phages and the following decrease of *Campylobacter* counts in that group (grey and black line in Figure 2). Therefore, only the first measurements are expedient for a solid evaluation of phage treatment efficiency. In trial II, the *Campylobacter* level of both treated groups is generally below the level of the control, except day three after application of the single phage (Figure 3b). The difference is significant (p< 0.001 - 0.036) from 7 days to 35 days after application for both treatment groups. The largest reduction of log_10_ 2.8 CFU/g cecal contents was measured on day 21.

### Resistant and less susceptible *Campylobacter* subpopulation

#### Scope of investigations

A total of 1,540 *Campylobacter* isolates from trial I were tested for their phage susceptibility to each phage and the phage cocktail. 2,640 isolates from trial II were tested for resistance to the phage cocktail generally and additionally to the individual phages they were subjected to.

#### Progression of reduced phage susceptibility

**Figure 4:**
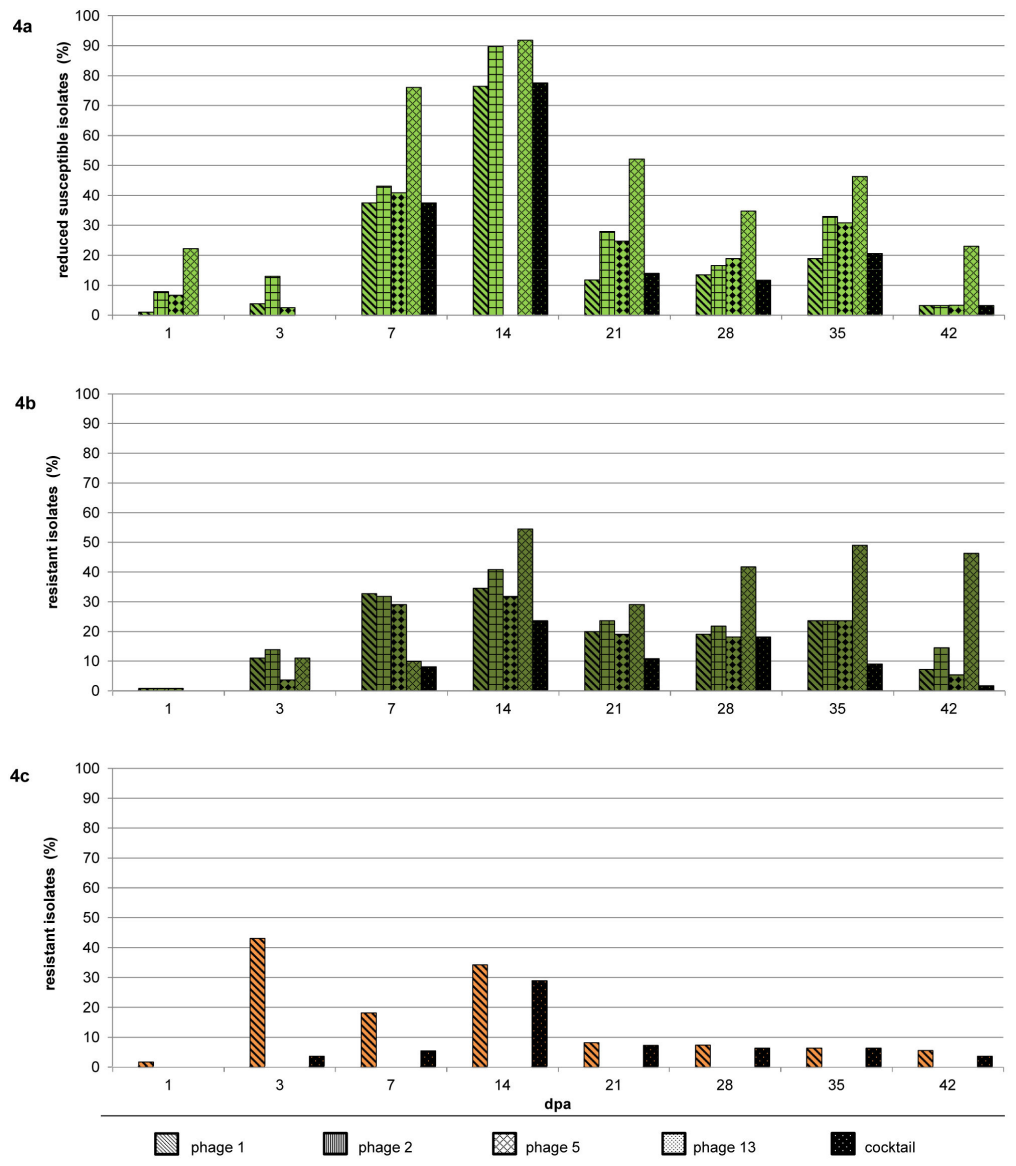
Emergence of less susceptible/resistant *Campylobacter* isolates. Part A shows the percentage of isolates from the cocktail treated group of trial I with reduced susceptibility against the individual phages and the cocktail. Data for phage 5 on day 14 after treatment are lacking. Part B shows the percentage of isolates from the cocktail treated group of trial II that are resistant to infection by the individual phages and the cocktail. Part C shows the percentage of isolates from the single phage treated group of trial II that are resistant to infection by phage 1 or the cocktail. Resistance for phages 2, 5 and 13 was not separately tested for this group.

In all phage treated groups a similar course of the emergence of less susceptible or resistant isolates was observed. Generally, phage susceptibility decreased first and subsequently increased towards the end of the investigation period (Figure 4a-c). One day after phage application, there were only slight changes in phage susceptibility of the *Campylobacter* population in all treated groups. Then, phage susceptibility decreased over two weeks after application of the phage cocktail (Figure 4a+b), but only three days after treatment with the single phage (Figure 4c). Towards the end of examinations, the proportion of the phage susceptible *Campylobacter* population increased again and reached 98% (cocktail) or 94% (phage 1), respectively.

In trial I up to ninety percent of the isolates from the cocktail treated group were less susceptible or resistant two weeks after phage application (Figure 4a). For this time point, the results for phage 5 are not given, due to technical faults. The susceptibility for the cocktail followed the same course as observed for the single phages. Only loss of susceptibility for phage 13 started faster and decreased slower.

In the cocktail treated group of trial II (Figure 4b), the course of resistance to phages 1, 2 and 5 was nearly identical and correlated well with the course observed in trial I. Resistance to phage 13 stayed at about 50% towards the end. Resistance to the cocktail was less common than resistance to the individual phages.

In the single phage treated group (Figure 4c) resistance to phage 1 arose earlier reaching 43 %, then dropped back earlier but finally resulted in the same level compared to the cocktail treated groups. Also cross-resistance to the phage cocktail was observed, following a similar trend observed in the cocktail treated groups. From day 21 after phage application the course of resistance to phage 1 and the cocktail were nearly identical in that group.

The phage infected control group of trial I showed a similar emergence of a less susceptible *Campylobacter* subpopulation as seen in the treatment group (bars in Figure 2). In contrast, only loss of susceptibility for phages 1, 2 and 5 was observed, phage 13 remained fully efficacious with all examined isolates. The highest occurrence of less susceptible isolates was approximately 70%. The following decline of the less susceptible part of the *Campylobacter* population was lower as observed in the treatment group. The emergence of a less susceptible subpopulation correlated with the detection of phages and the decline of the *Campylobacter* counts in that group.

#### Total resistance to the single phages and the phage cocktail, considering the different treatments

Table 2 shows for each group how many *Campylobacter* isolates were less susceptible (trial I) or resistant (trial II) in total for the several phages during the investigation period. 

**Table 2:** Percentage of all *Campylobacter* isolates examined that are less susceptible (trial I) or resistant (trial II) to the individual phages and the cocktail.

trial	group	phage 1	phage 2	phage 5	phage 13	cocktail
trial I	cocktail	20.83	29.34	16.43	43.33	20.58
	infected control	24.86	35.06	33.87	0.00	0.13
trial II	cocktail	18.66	21.40	16.49	30.25	8.98
	phage 1	15.61	-	-	-	7.73
	control	-	-	-	-	0.23

Loss of susceptibility or increase of resistance was the most pronounced for phage 13 and the least pronounced for phage 5, regarding the individual phages; the cocktail was generally less affected.

The phages which infected the control group of trial I induced a stronger loss of susceptibility for phages 1, 2 and 5 than the experimentally administered phage cocktail, but no loss of susceptibility for phage 13.

The cocktail treatment in trial II induced marginally more resistance to phage 1 and the cocktail than treatment with only phage 1 did.

Only two out of 880 *Campylobacter* isolates from the phage free control group of trial II showed spontaneous resistance to the phage cocktail. Both were isolated from one bird.

### Impact on *Campylobacter* colonization level

**Figure 5:**
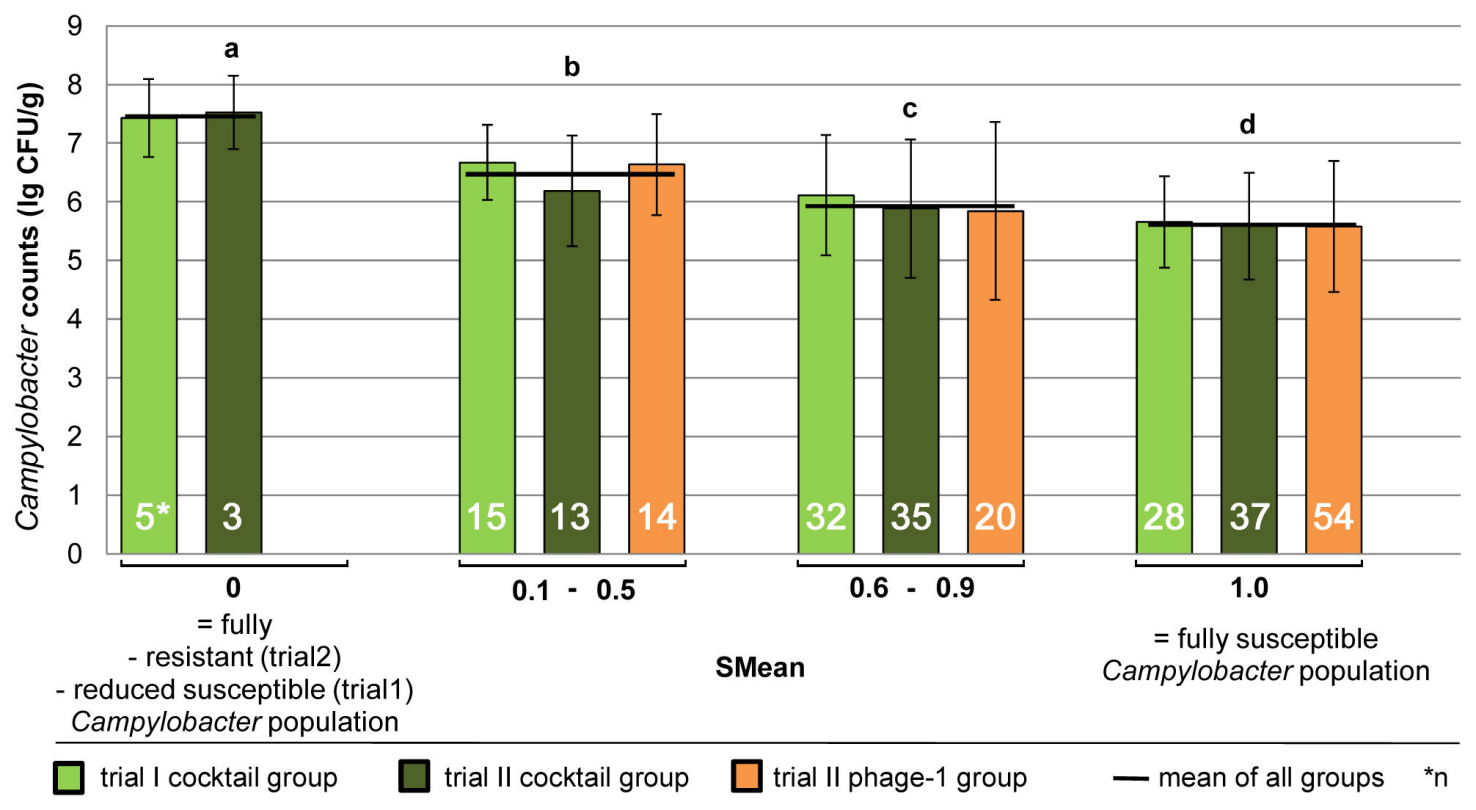
*Campylobacter* counts of birds harboring different amounts of phage resistant bacteria. All birds from the treated groups were categorized into four groups, depending on the amount of phage resistant *Campylobacter* isolates in their ceca (measured as SMean).

**Figure 6:**
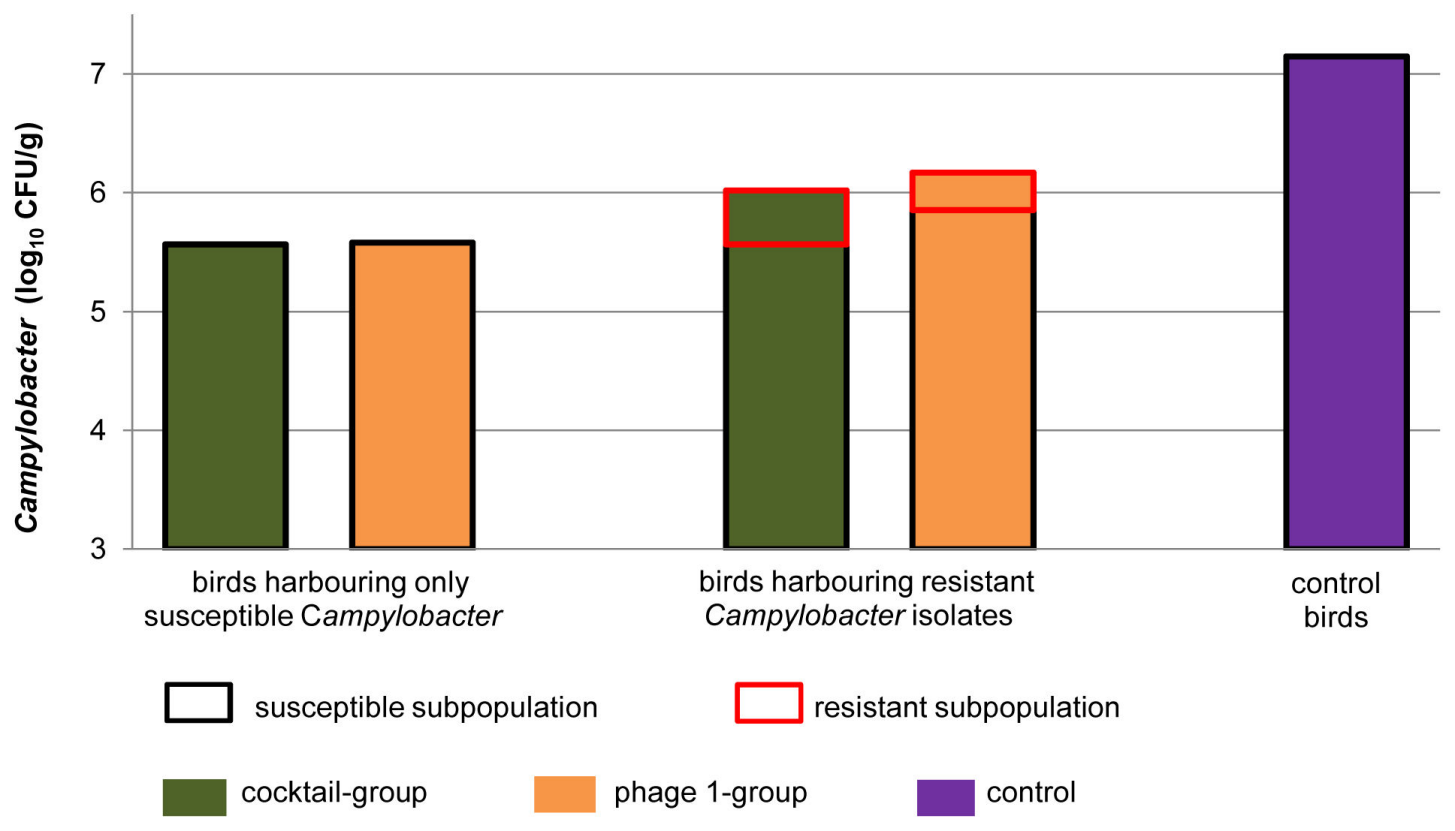
Influence of phage resistant bacteria on the reduction potential of phages. Comparison of the mean *Campylobacter* level of i) treated birds harboring a pure susceptible *Campylobacter* population ii) treated birds harboring resistant isolates and iii) untreated birds. For the birds harbouring resistant isolates (ii) two mean counts were calculated: the mean of the *Campylobacter* counts, including the resistant isolates and the mean of the *Campylobacter* counts after deducting the resistant subpopulation.

The interrelation between the degree of resistance of one bird´s *Campylobacter* population and its *Campylobacter* colonization level is presented in figures 5 and 6.

A good correlation (Figure 5) exists between the proportion of the resistant *Campylobacter* subpopulation, measured as “Susceptibility Mean” (SMean), and the *Campylobacter* colonization level. Birds whose *Campylobacter* population was categorized into the lowest susceptible group of SMean=0 were colonized the most at log_10_ 7.5 CFU/g on average. The *Campylobacter* colonization level decreased continuously with rising susceptibility and reached log_10_ 5.6 CFU/g at an SMean of 1.

Therefore, the *Campylobacter* colonization level in phage treated birds, harboring a resistant or less susceptible *Campylobacter* subpopulation, was higher (log_10_ 6.16 CFU/g cecal contents on average; Figure 6) compared to the level of phage treated birds harboring a pure susceptible *Campylobacter* population (log_10_ 5.56 CFU/g cecal contents), but did not reach the colonization level of the phage free control birds (log_10_ 7.15 CFU/g cecal contents). After deducting the subpopulation showing resistance or reduced susceptibility, a colonization level of log_10_ 5.65 CFU/g for the susceptible *Campylobacter* subpopulation of mixed colonized birds resulted. Therefore, the mean colonization level, reached by a susceptible *Campylobacter* population was about log_10_ 5.61 CFU/g. The mean increase caused by the *Campylobacter* subpopulation showing resistance or reduced susceptibility was estimated to be about log_10_ 0.51 CFU/g.

## Discussion

### Current framework conditions

Neither the overall prevalence of *Campylobacter* in chicken retail products, nor the number of reported poultry meat consumption-related human campylobacteriosis cases have been reduced in recent years [23,24]. It is difficult to keep broiler farms free from *Campylobacter* colonization because small numbers of the ubiquitous and well adapted bacteria suffice to colonize the birds´ intestine [25]. *Campylobacter* enters the food chain continuously on a farm level and survives all stages from ´farm to fork` [5,26,27]. Therefore, all stages from enhanced prevention measures at farm level to improved consumer information have been considered and evaluated for their suitability as critical control points to reduce the risk of human campylobacteriosis [4–7,28–31]. Reduction of contamination at broiler farms is considered to be efficient, but a major decrease of infections at a farm level is not expected in the short term [30] as there is still no effective, reliable and practical intervention measure available to prevent or to reduce *Campylobacter* colonization in poultry [6,32]. Three general strategies have been proposed to control *Campylobacter* in poultry at farm level: i) reduction of environmental exposure, ii) an increase in poultry’s host resistance to reduce *Campylobacter* carriage in the gut, and iii) the use of antimicrobial alternatives to reduce and even eradicate *Campylobacter* from colonized chickens [32]. Regarding the growing number of organic and free range flocks having a very high risk of becoming *Campylobacter* positive by contamination with bacteria from the environment [33], the bacterial load reducing measures including bacteriophages are becoming increasingly important.

### Phage load

Phages are found in high numbers, provided that their hosts occur, in seawater as well as on food [34]. Just a few studies have evaluated the natural incidence of *Campylobacter* phages in poultry and on poultry products. Phages could be isolated from 3% up to 51% of the samples examined, whereby free range husbandries were more frequently affected compared to indoor systems [10,12,35–38]. A wide range of bacteriophage titers from log_10_ 1.5 to log_10_ 6.9 PFU/g cecal contents of the individual birds were recovered in these studies [10,12]. Also, several studies concerning the experimental phage treatment of *Campylobacter* in broilers and considering the phage titers were conducted [13–16]. All the treatments of *Campylobacter* colonized chickens resulted in the bacteriophages persisting and replicating in the chickens` intestinal tract. Wagenaar et al. [15] found opposing highs and lows of *Campylobacter* and phage counts after several repeated phage applications and interpreted this as alternating shifts in amplification of bacteria and phages. The mean phage counts of *Campylobacter* colonized birds ranged from log_10_ 5 to log_10_ 8 PFU/g faeces, whereby the counts tended to decrease after the end of the treatment. Loc Carillo et al. [16] recovered phage titers ranging from log_10_ 3.2 to log_10_ 6.5 PFU/g cecal contents which did not correlate with simple dilutions of the different input doses (log_10_ 5, 7 and 9 PFU/bird), implying that viral replication occurred within 24 h of administration. El Shibiny et al. [14] likewise found that irrespective of the initial phage dose administered, the mean phage counts stabilized at certain levels between log_10_ 4 and log_10_ 5.9 PFU/g intestinal contents depending on the intestinal segment. Carvalho et al. [13] found different mean phage counts after administering the same phage cocktail to *Campylobacter jejuni* (log_10_ 5.3 PFU/g feces) and to *Campylobacter coli* (log_10_ 3.4 PFU/g feces) colonized birds. Nevertheless, these phage counts remained constant throughout the experimental period of seven days within both groups. The mean phage titers, obtained in our experiments ranging from log_10_ 2.7 PFU/g to log_10_ 6.1 PFU/g are in accordance with previously reported phage counts of naturally occurring and experimentally administered phages in the birds´ intestine. We also found the bacteriophages replicating and existing at stable levels at about log_10_ 4 PFU/g over the whole investigation period of six weeks (Figure 1), which is in accordance with other studies [13–16]. It seems that the present *Campylobacter* and phage strain combinations result in a clear natural balance. In contrast to the previously reported initial high phage titers, we found a remarkably low level of phage 1 one day after application. This might be explained by a low pH tolerance of the phage, which has been reported for several other phages [14,39]. Furthermore, this could also explain why the phages did not reach the intestine of some birds and why the phages excreted by the phage positive birds did not suffice to allow self-treatment after orally intaking of excrements over at least six weeks.

### *Campylobacter* load and reduction

The considerable variation of *Campylobacter* titers from the individual birds was previously described [40]. Different studies revealed the effect of phages on the *Campylobacter* counts in chickens [10–16,41]. The presence of phages in various commercial flocks resulted in an on average statistically significant log_10_ 1.8 CFU/g lower *Campylobacter* load compared to the phage free flocks in that study [10]. Different treatment designs including water and feed treatment, different dosages, preventive and therapeutic applications, single and repeated administration, using different phage and *Campylobacter* strains have been tested for their potential to reduce *Campylobacter* in chickens. These studies are inter alia reviewed by Connerton et al. [42]. Reductions of the *Campylobacter* load up to log_10_ 5.6 CFU/g intestinal contents have been found. Preventive gavage of phages delayed the colonization of *Campylobacter*. Uptake of phages incorporated in feed resulted in earlier reduction of *Campylobacter* compared to oral gavage. Several times a prominent initial drop of the *Campylobacter* load, followed by a re-increase of the *Campylobacter* level, resulting in a poor reduction level compared to the controls, was detected [15,16]. Therefore, a treatment of the flocks shortly before slaughter has been suggested [13]. In contrast to that, our results show an initial low reduction, followed by a four-week period of significant reduction and ending again with low reduction levels (Figure 3b). Therefore, an adequate timeframe of between one and four weeks before slaughter would be reasonable. Nevertheless, the treatment shortly before slaughter would be more practical, since *Campylobacter* can enter the flock towards the very end of fattening. An early phage gavage would then be useless, since phages can only persist and replicate in the intestine in presence of their host [15].

Phage cocktails were assembled in order to enlarge the host range. In addition, we looked for benefits of the cocktail concerning the reduction potential and emergence of resistance. In trial II the cocktail administration resulted in a basic reduction, whereas the administration of the single phage could not maintain the usually observed reduction on day three after treatment (Figure 3b). This coincided with the early short term emergence of the highest resistance rate of the *Campylobacter* population (Figure 4c, phage 1 bar). The combination with three further phages resulted in a delayed and lower emergence of isolates resistant to the applied cocktail (Figure 4b, cocktail bar). Here, we compare resistance to phage 1 in the single phage group and resistance to the cocktail in the cocktail group since the resistance to the entity of the applied and therapeutically active phages is decisive for the efficiency of the treatment.

### Resistances

#### Impact of phage resistance on *Campylobacter* colonization level

Resistances are seen as a possible obstacle for short- and long-term phage treatment efficiency. When phages were previously used to reduce *Campylobacter* counts in the intestine of poultry, phage resistant *Campylobacter* isolates were observed. Resistance rates from 2% [14] up to 13% [13] were reported. Even the impact of resistant *Campylobacter* isolates to the following phage treatment was examined. Loc Carrillo et al. [16] found a significantly reduced capability of a phage resistant isolate to colonize the broiler intestine compared to the susceptible original strain, whereas Carvalho et al. [13] could not confirm such a difference in comparable studies. The findings of investigations concerning the preservation of resistance during a further intestinal passage differ highly. The percentage of isolates, which lost their resistant phenotype, was specified by the mentioned authors as being between 54% and 97%. In order to avoid an accumulation of phage resistant *Campylobacter* strains in the farm environment it has been proposed to limit the use of phages to the abattoirs [19].

It seems that the phages used in this study kept the colonization level of the susceptible *Campylobacter* 1474-06 population at approximately 5.6 CFU/g (Figure 6). Once resistant subpopulations occurred they were not subjected to the reduction by phages and increased the total *Campylobacter* load without reaching the colonization level of the phage free control birds when considering the trials as a whole. However, the greater amount of the resistant *Campylobacter* population of some birds correlated with higher *Campylobacter* loads (Figure 5). The inability of the resistant subpopulation to maintain the colonization level of the control birds over time might be due to a reduced colonization capability of the resistant isolates, which is described as accompanying resistance [16].

Regarding these findings and especially the increase and decrease of the phage resistant subpopulation, we assume that the previously described different outcomes not only depend on different phage-bacteria combinations but also on the different time points of investigations (Figure 4). Furthermore, our findings suggest that long term efficiency is not seriously compromised by the emergence of resistance. Therefore, we agree with Wagenaar et al. [15], who consider the release of phages into the environment to be acceptable. Nevertheless, this must be validated for more phage bacteria combinations, considering additionally the resistance mechanisms in order to develop well designed phage bio-control strategies for the enhancement of food safety.

#### Cross-resistances in the single phage treated group

In the single phage treated group first resistance to the deployed phage arose, followed by resistance to the whole phage cocktail (Figure 4c). Therefore, it seems that different resistance mechanisms occurred, first a phage 1 specific mechanism and afterwards a more basic mechanism, perhaps changing the structure required for phage bacteria interaction, resulting in cross resistance to the three other phages tested. The resistance mechanism should be specified in further studies. At the moment, this underlines the importance of well-founded knowledge about phage bacteria interactions in order to combine different acting phages to wide ranging and resistance avoiding cocktails.

#### Spontaneous resistance in the control group of trial II

Spontaneous resistance of *Campylobacter* 1474-06 to the four phages *in vitro* was not observed in already published examinations [20]. Nevertheless, after colonization in the broiler gut two spontaneous resistant isolates were recovered (table 2). Carvalho et al. [13] observed 6% resistant *Campylobacter jejuni* isolates from untreated chickens inoculated with a susceptible *Campylobacter* strain as well. The amount of bacteria and the multiplication frequency is considerably higher in the gut environment compared to the *in vitro* environment. Therefore, it was not surprising that the naturally occurring resistant strains [43] were recovered from the cecal and not from the *in vitro* samples.

### Infected control group

This group was included in this paper despite infection in order to offer the data from a fresh natural phage infection. We assumed an infection by one or more unknown phages from the environment due to the different susceptibility pattern: Significant lower phage levels compared to the treatment groups were found, which may be the strongest indication for an infection by an unknown phage. Furthermore, no loss of susceptibility for phage 13 was observed, in contrast to development of cross-resistance to all phages, including phage 13 in the single phage treated group of trial II. Therefore, an infection by phage 1 is unlikely, but we cannot obviously rule out that phages 2, 5 or 13 were transferred.

## Conclusion

Bacteriophages are able to reduce the *Campylobacter* load in chicken sustainably. We could show for the first time that resistances of *Campylobacter* against phages stabilized at a low level after an initial increase, which could be moderated and delayed by the use of more than one phage. Considering our own and previously described findings [10–16] we believe that phage bio-control could play a promising role in combating *Campylobacter* at farm level and thus in reducing human campylobacteriosis. Intelligent combinations of different phages could contribute to the success of phage bio-control by reaching a broad host range and keeping resistances under control. For this, further studies are required in order to gain sufficient knowledge of phage-bacteria interactions of all phages intended for future commercial use.

## References

[1] ECDC (2011) Annual epidemiological report 2011. Eurosurveillance 16: 17-17.

[2] Louwen R, Horst-Kreft D, de Boer AG, van der Graaf L, de Knegt G et al. (2012) A novel link between *Campylobacter* *jejuni* bacteriophage defence, virulence and Guillain-Barre syndrome. European journal of clinical microbiology & infectious diseases : official publication of the European Society of Clinical Microbiology. 10.1007/s10096-012-1733-4 22945471

[3] EFSA (2011) Scientific opinion on *Campylobacter* in broiler meat production: control options and performance objectives and/or targets at different stages of the food chain. EFSA J 9: 2105.

[4] Lammerding AM, Fazil A (2000) Hazard identification and exposure assessment for microbial food safety risk assessment. Int J Food Microbiol 58: 147-157. doi:10.1016/S0168-1605(00)00269-5. PubMed: 10939265. 10939265

[5] Rosenquist H, Nielsen NL, Sommer HM, Nørrung B, Christensen BB (2003) Quantitative risk assessment of human campylobacteriosis associated with thermophilic *Campylobacter* species in chickens. Int J Food Microbiol 83: 87-103. doi:10.1016/S0168-1605(02)00317-3. PubMed: 12672595. 12672595

[6] Ghareeb K, Awad WA, Mohnl M, Schatzmayr G, Bohm J (2013) Control strategies for *Campylobacter* infection in poultry production. Worlds Poult Sci J 69: 57-76. doi:10.1017/S0043933913000068.

[7] Pasquali F, De Cesare A, Manfreda G, Franchini A (2011) *Campylobacter* control strategies in european poultry production. Worlds Poult Sci J 67: 5-18. doi:10.1017/S0043933911000018.

[8] Üffing B (2011) Einfluss der Mischfutterherstellung (Art der Vermahlung/ Konfektionierung) auf ausgewählte Keimgruppen der Gastrointestinalflora von Masthähnchen. Hannover: Stiftung Tierärztliche Hochschule Hannover. 203 p

[9] Atterbury RJ, Connerton PL, Dodd CE, Rees CE, Connerton IF (2003) Isolation and characterization of *Campylobacter* bacteriophages from retail poultry. Appl Environ Microbiol 69: 4511-4518. doi:10.1128/AEM.69.8.4511-4518.2003. PubMed: 12902236. 12902236 PMC169066

[10] Atterbury RJ, Dillon E, Swift C, Connerton PL, Frost JA et al. (2005) Correlation of *Campylobacter* bacteriophage with reduced presence of hosts in broiler chicken ceca. Appl Environ Microbiol 71: 4885-4887. doi:10.1128/AEM.71.8.4885-4887.2005. PubMed: 16085889. 16085889 PMC1183290

[11] Connerton PL, Loc Carrillo CM, Swift C, Dillon E, Scott A et al. (2004) Longitudinal study of *Campylobacter* *jejuni* bacteriophages and their hosts from broiler chickens. Appl Environ Microbiol 70: 3877-3883. doi:10.1128/AEM.70.7.3877-3883.2004. PubMed: 15240258. 15240258 PMC444807

[12] El-Shibiny A, Connerton PL, Connerton IF (2005) Enumeration and diversity of *Campylobacters* and bacteriophages isolated during the rearing cycles of free-range and organic chickens. Appl Environ Microbiol 71: 1259-1266. doi:10.1128/AEM.71.3.1259-1266.2005. PubMed: 15746327. 15746327 PMC1065130

[13] Carvalho CM, Gannon BW, Halfhide DE, Santos SB, Hayes CM et al. (2010) The in vivo efficacy of two administration routes of a phage cocktail to reduce numbers of *Campylobacter* *coli* and *Campylobacter* *jejuni* in chickens. BMC Microbiol 10: 232. doi:10.1186/1471-2180-10-232. PubMed: 20809975. 20809975 PMC2940857

[14] El-Shibiny A, Scott A, Timms A, Metawea Y, Connerton P et al. (2009) Application of a group II *Campylobacter* bacteriophage to reduce strains of *Campylobacter* *jejuni* and *Campylobacter* *coli* colonizing broiler chickens. J Food Protect 72: 733-740. PubMed: 19435220. 10.4315/0362-028x-72.4.733 19435220

[15] Wagenaar JA, Van Bergen MAP, Mueller MA, Wassenaar TM, Carlton RM (2005) Phage therapy reduces *Campylobacter* *jejuni* colonization in broilers. Vet Microbiol 109: 275–283. doi:10.1016/j.vetmic.2005.06.002. PubMed: 16024187. 16024187

[16] Loc Carrillo C, Atterbury RJ, el-Shibiny A, Connerton PL, Dillon E et al. (2005) Bacteriophage therapy to reduce *Campylobacter* *jejuni* colonization of broiler chickens. Appl Environ Microbiol 71: 6554-6563. doi:10.1128/AEM.71.11.6554-6563.2005. PubMed: 16269681. 16269681 PMC1287621

[17] Coffey B, Mills S, Coffey A, McAuliffe O, Ross RP (2010) Phage and their lysins as biocontrol agents for food safety applications. Annu Rev Foods Science Technol 1: 449-468. doi:10.1146/annurev.food.102308.124046. PubMed: 22129344. 22129344

[18] Glünder G, Windhaus H (1998) Investigtation on *Campylobacter* in turkeys. Proc 1st international Symposium on Turkey Diseases. 19.-21 02.1998 ed. Berlin: Verlag der deutschen Veterinärmedizinischen Gesellschaft e.V. pp. 307-316

[19] Goode D, Allen VM, Barrow PA (2003) Reduction of experimental *Salmonella* and *Campylobacter* contamination of chicken skin by application of lytic bacteriophages. Appl Environ Microbiol 69: 5032-5036. doi:10.1128/AEM.69.8.5032-5036.2003. PubMed: 12902308. 12902308 PMC169133

[20] Fischer S, Kittler S, Klein G, Glünder G (2013) Microplate-test for the rapid determination of bacteriophage-susceptibility of *Campylobacter* isolates-development and validation. PLOS ONE 8: e53899. doi:10.1371/journal.pone.0053899. PubMed: 23349761. 23349761 PMC3547971

[21] Hirsch K (2010) Development of strategies for minimizing *Campylobacter* in poultry by utilizing bacteriophages. Vet. med. Thesis, University of Vet. Med., Hannover: University of Veterinary Medicine Hannover, Foundation

[22] Marshall SM, Melito PL, Woodward DL, Johnson WM, Rodgers FG et al. (1999) Rapid identification of *Campylobacter,* *Arcobacter*, and *Helicobacter* isolates by PCR-restriction fragment length polymorphism analysis of the 16S rRNA gene. J Clin Microbiol 37: 4158-4160. PubMed: 10565952. 10565952 10.1128/jcm.37.12.4158-4160.1999 PMC85910

[23] Moran L, Scates P, Madden RH (2009) Prevalence of *Campylobacter* spp. in raw retail poultry on sale in Northern Ireland. J Food Protect 72: 1830-1835. PubMed: 19777882. 10.4315/0362-028x-72.9.1830 19777882

[24] EFSA (2012) The European Union summary report on trends and sources of zoonoses, zoonotic agents and food-borne outbreaks in 2010. EFSA J 10: 2597. 22433599

[25] Ogden ID, Dallas JF, MacRae M, Rotariu O, Reay KW et al. (2009) *Campylobacter* excreted into the environment by animal sources: prevalence, concentration shed, and host association. Foodborne Pathog Dis 6: 1161-1170. doi:10.1089/fpd.2009.0327. PubMed: 19839759. 19839759 PMC3985071

[26] Reich F, Atanassova V, Haunhorst E, Klein G (2008) The effects of *Campylobacter* numbers in caeca on the contamination of broiler carcasses with *Campylobacter* . Int J Food Microbiol 127: 116-120. doi:10.1016/j.ijfoodmicro.2008.06.018. PubMed: 18657873. 18657873

[27] Ellerbroek LI, Lienau JA, Klein G (2010) *Campylobacter* spp. in broiler flocks at farm level and the potential for cross-contamination during slaughter. Zoonoses Public Health 57: E81-E88. doi:10.1111/j.1863-2378.2009.01267.x. PubMed: 20880094. 20880094

[28] Wagenaar JA, Mevius DJ, Havelaar AH (2006) *Campylobacter* in primary animal production and control strategies to reduce the burden of human campylobacteriosis. Rev Sci Tech-Off Int Epizoot 25: 581-594. PubMed: 17094699. 17094699

[29] Osiriphun S, Iamtaweejaloen P, Kooprasertying P, Koetsinchai W, Tuitemwong K et al. (2011) Exposure assessment and process sensitivity analysis of the contamination of *Campylobacter* in poultry products. Poult Sci 90: 1562-1573. doi:10.3382/ps.2009-00577. PubMed: 21673173. 21673173

[30] Havelaar AH, Mangen MJJ, de Koeijer AA, Bogaardt MJ, Evers EG et al. (2007) Effectiveness and efficiency of controlling *Campylobacter* on broiler chicken meat. Risk Anal 27: 831-844. doi:10.1111/j.1539-6924.2007.00926.x. PubMed: 17958495. 17958495

[31] Hermans D, Van Deun K, Messens W, Martel A, Van Immerseel F et al. (2011) *Campylobacter* control in poultry by current intervention measures ineffective: urgent need for intensified fundamental research. Vet Microbiol 152: 219-228. doi:10.1016/j.vetmic.2011.03.010. PubMed: 21482043. 21482043

[32] Lin J (2009) Novel approaches for *Campylobacter* control in poultry. Foodborne Pathog Dis 6: 755-765. doi:10.1089/fpd.2008.0247. PubMed: 19425824. 19425824 PMC3145176

[33] Näther G, Alter T, Martin A, Ellerbroek L (2009) Analysis of risk factors for *Campylobacter* species infection in broiler flocks. Poult Sci 88: 1299-1305. doi:10.3382/ps.2008-00389. PubMed: 19439643. 19439643

[34] Chibani-Chennoufi S, Bruttin A, Dillmann ML, Brüssow H (2004) Phage-host interaction: an ecological perspective. J Bacteriol 186: 3677-3686. doi:10.1128/JB.186.12.3677-3686.2004. PubMed: 15175280. 15175280 PMC419959

[35] Hansen VM, Rosenquist H, Baggesen DL, Brown S, Christensen BB (2007) Characterization of *Campylobacter* phages including analysis of host range by selected *Campylobacter* Penner serotypes. BMC Microbiol 7: 90. doi:10.1186/1471-2180-7-90. PubMed: 17945022. 17945022 PMC2194700

[36] Heuer OE, Pedersen K, Andersen JS, Madsen M (2001) Prevalence and antimicrobial susceptibility of thermophilic *Campylobacter* in organic and conventional broiler flocks. Lett Appl Microbiol 33: 269-274. doi:10.1046/j.1472-765X.2001.00994.x. PubMed: 11559399. 11559399

[37] Hwang S, Yun J, Kim KP, Heu S, Lee S et al. (2009) Isolation and characterization of bacteriophages specific for *Campylobacter* *jejuni* . Microbiol Immunol 53: 559-566. doi:10.1111/j.1348-0421.2009.00163.x. PubMed: 19780969. 19780969

[38] Tsuei AC, Carey-Smith GV, Hudson JA, Billington C, Heinemann JA (2007) Prevalence and numbers of coliphages and *Campylobacter* *jejuni* bacteriophages in New Zealand foods. Int J Food Microbiol 116: 121-125. doi:10.1016/j.ijfoodmicro.2006.12.028. PubMed: 17276534. 17276534

[39] Ma YS, Pacan JC, Wang Q, Xu YP, Huang XQ et al. (2008) Microencapsulation of bacteriophage Felix O1 into chitosan-alginate microspheres for oral delivery. Appl Environ Microbiol 74: 4799-4805. doi:10.1128/AEM.00246-08. PubMed: 18515488. 18515488 PMC2519356

[40] Rudi K, Høidal HK, Katla T, Johansen BK, Nordal J et al. (2004) Direct real-time PCR quantification of *Campylobacter* *jejuni* in chicken fecal and cecal samples by integrated cell concentration and DNA purification. Appl Environ Microbiol 70: 790-797. doi:10.1128/AEM.70.2.790-797.2004. PubMed: 14766556. 14766556 PMC348921

[41] Scott AE, Timms AR, Connerton PL, El-Shibiny A, Connerton IF (2007) Bacteriophage influence *Campylobacter* *jejuni* types populating broiler chickens. Environ Microbiol 9: 2341-2353. doi:10.1111/j.1462-2920.2007.01351.x. PubMed: 17686030. 17686030

[42] Connerton PL, Timms AR, Connerton IF (2011) *Campylobacter* bacteriophages and bacteriophage therapy. J Appl Microbiol 111: 255-265. doi:10.1111/j.1365-2672.2011.05012.x. PubMed: 21447013. 21447013

[43] Luria SE, Delbrück M (1943) Mutations of bacteria from virus sensitivity to virus resistance. Genetics 28: 491-511. PubMed: 17247100. 17247100 10.1093/genetics/28.6.491 PMC1209226

